# Author Correction: The IPDGC/GP2 Hackathon - an open science event for training in data science, genomics, and collaboration using Parkinson’s disease data

**DOI:** 10.1038/s41531-023-00529-6

**Published:** 2023-05-24

**Authors:** Hampton L. Leonard, Ruqaya Murtadha, Alejandro Martinez-Carrasco, Alina Jama, Amica Corda Müller-Nedebock, Ana-Luisa Gil-Martinez, Anastasia Illarionova, Anni Moore, Bernabe I. Bustos, Bharati Jadhav, Brook Huxford, Catherine Storm, Clodagh Towns, Dan Vitale, Devina Chetty, Eric Yu, Francis P. Grenn, Gabriela Salazar, Geoffrey Rateau, Hirotaka Iwaki, Inas Elsayed, Isabelle Francesca Foote, Zuné Jansen van Rensburg, Jonggeol Jeff Kim, Jie Yuan, Julie Lake, Kajsa Brolin, Konstantin Senkevich, Lesley Wu, Manuela M. X. Tan, María Teresa Periñán, Mary B. Makarious, Michael Ta, Nikita Simone Pillay, Oswaldo Lorenzo Betancor, Paula R. Reyes-Pérez, Pilar Alvarez Jerez, Prabhjyot Saini, Rami al-Ouran, Ramiya Sivakumar, Raquel Real, Regina H. Reynolds, Ruifneg Hu, Shameemah Abrahams, Shilpa C. Rao, Tarek Antar, Thiago Peixoto Leal, Vassilena Iankova, William J. Scotton, Yeajin Song, Andrew Singleton, Mike A. Nalls, Sumit Dey, Sara Bandres-Ciga, Cornelis Blauwendraat, Alastair J. Noyce, on behalf of The International Parkinson Disease Genomics Consortium (IPDGC) and The Global Parkinson’s Genetics Program (GP2)

**Affiliations:** 1 Laboratory of Neurogenetics, National Institute on Aging, National Institutes of Health, Bethesda, MD USA; 2 Center for Alzheimer’s and Related Dementias (CARD), National Institute on Aging and National Institute of Neurological Disorders and Stroke, National Institutes of Health, Bethesda, MD USA; 3 Data Tecnica International LLC, Washington, DC USA; 4 German Center for Neurodegenerative Diseases (DZNE), Tübingen, Germany; 5 Department of Clinical and Movement Neurosciences, UCL Queen Square Institute of Neurology, University College London, London, UK; 6 Department of Neuromuscular Diseases, UCL Queen Square Institute of Neurology, University College London, London, UK; 7 Division of Molecular Biology and Human Genetics, Department of Biomedical Sciences, Faculty of Medicine and Health Sciences, Stellenbosch University, Cape Town, South Africa; 8 South African Medical Research Council/Stellenbosch University Genomics of Brain Disorders Research Unit, Stellenbosch University, Cape Town, South Africa; 9 Department of Neurodegenerative Disease, University College London, London, UK; 10 Great Ormond Street Institute of Child Health, Genetics and Genomic Medicine, University College London, London, UK; 11 The Ken & Ruth Davee Department of Neurology and Simpson Querrey Center of Neurogenetics, Feinberg Feinberg School of Medicine, Northwestern University, Chicago, IL 60611 USA; 12 Department of Genetics and Genomic Sciences and Mindich Child Health and Development Institute, Icahn School of Medicine at Mount, Hess Center for Science and Medicine, New York, NY 10029 USA; 13 Preventive Neurology Unit, Wolfson Institute of Population Health, Queen Mary University of London, London, UK; 14 Department of Human Genetics, McGill University, Montreal, QC Canada; 15 The Neuro (Montreal Neurological Institute-Hospital), McGill University, Montreal, QC Canada; 16 INNCOSYS, Col. Morelos Second Section, 50120 Toluca de Lerdo, México; 17 Institut du Cerveau - Institute of Brain and Spine (ICM), Hôpital Pitié, 47 Bd de l’Hôpital, 75013 Paris, France; 18 Faculty of pharmacy, University of Gezira, Wad Medani, P.O. Box 20, Sudan; 19 International Parkinson Disease Genomics Consortium (IPDGC)-Africa, University of Gezira, Wad Medani, P.O. Box 20, Sudan; 20 Unit for Psychological Medicine, Wolfson Institute of Population Health, Queen Mary University of London, London, UK; 21 Center for Advanced Parkinson Research, Brigham and Women’s Hospital, Harvard Medical School, Boston, MA 02115 USA; 22 Translational Neurogenetics Unit, Wallenberg Neuroscience Center, Department of Experimental Medical Science, Lund University, Lund, Sweden; 23 Department of Neurology and Neurosurgery, McGill University, Montréal, QC Canada; 24 Department of Neurology, Oslo University Hospital, Oslo, Norway; 25 Unidad de Trastornos del Movimiento, Servicio de Neurología y Neurofisiología Clínica, Instituto de Biomedicina de Sevilla, Hospital Universitario Virgen del Rocío/CSIC/Universidad de Sevilla, Seville, Spain; 26 CIBERNED, Madrid, Spain; 27 South African National Bioinformatics Institute (SANBI), South African Medical Research Council Bioinformatics Unit, University of the Western Cape, Bellville, South Africa; 28 Veterans Affairs Puget Sound Health Care System, Seattle, WA USA; 29 Department of Neurology, University of Washington School of Medicine, Seattle, WA USA; 30 Laboratorio Internacional de Investigación sobre el Genoma Humano, Universidad Nacional Autónoma de, México, Juriquilla México; 31 Department of Pediatrics, Baylor College of Medicine, Houston, TX 77030 USA; 32 University of Southern California, Los Angeles, CA 90007 USA; 33 Department of Genomic Medicine, Lerner Research Institute, Cleveland Clinic Foundation, Cleveland, OH 44195 USA; 34 Department of Molecular Medicine, Case Western Reserve University, Cleveland, OH 44106 USA; 35 Department of Neurology With Friedrich Baur Institut, University Hospital of Ludwig-Maximilians-Universität München, Munich, Germany

**Keywords:** Conferences and meetings, Genomics

Correction to: *npj Parkinson’s Disease* 10.1038/s41531-023-00472-6, published online 04 March 2023

In this article the affiliation details for Alastair J Noyce, Jonggeol Jeff Kim, Isabelle Francesca Foote, Sumit Dey were incorrectly given as ‘Department of Genetics and Genomic Sciences and Mindich Child Health and Development Institute, Icahn School of Medicine at Mount, Hess Center for Science and Medicine, New York, NY 10029, USA,’ but should have been ‘Preventive Neurology Unit, Wolfson Institute of Population Health, Queen Mary University of London, London, UK’.

The affiliation details for Prabhjyot Saini were incorrectly given as ‘Preventive Neurology Unit, Wolfson Institute of Population Health, Queen Mary University of London, London, UK’ but should have been ‘The Neuro (Montreal Neurological Institute-Hospital), McGill University, Montreal, QC, Canada’. The original article has been corrected.

