# Supplementary material for: The IPDGC/GP2 Hackathon - an open science event for training in data science, genomics, and collaboration using Parkinson’s disease data
Source: NPJ Parkinsons Dis. 2023 Mar 4;9:33. doi: 10.1038/s41531-023-00472-6 (PMC9984758; doi:10.1038/s41531-023-00472-6)
Supplement: Supplementary file 1 — Supplemental Material [file 41531_2023_472_MOESM1_ESM.pdf]

Name	Country	Institution
**Emilia Gatto**	Argentina	Institute of Neurosciences Buenos Aires
**Marcelo Kauffman**	Argentina	Ramos Mejia Hospital
**Julie Hunter**	Australia	ANZAC Research Institute
**Kishore Kumar**	Australia	University Sydney
**Miguel E. Renteria**	Australia	QIMR Berghofer Medical Research Institute
**Sulev Koks**	Australia	Murdoch University
**Alexander Zimprich**	Austria	Medical University of Vienna
**Artur Francisco Schumacher Schuh**	Brazil	Universidade Federal do Rio Grande do Sul
**Bruno Santos**	Brazil	Hospital Ophir Loyola
**Carlos Rieder**	Brazil	Alegre
**Vitor Tumas**	Brazil	University of São Paulo
**Oury Monchi**	Canada	University of Calgary
**Ted Fon**	Canada	McGill University
**Marcelo David Miranda**	Chile	Fundación Diagnosis
**Bustamante**	Chile	Fundación Diagnosis
**Patricio Olguin**	Chile	University of Chile
**Pedro Chana**	Chile	Corporación CETRAM
**Beisha Tang**	China	Central South University
**Hui-Fang Shang**	China	Sichuan University
**Ji Feng Guo**	China	Central South University
**Piu Chan**	China	Capital Medical University
**Wei Luo**	China	Zhejiang University
**Gonzalo Arboleda**	Colombia	National University of Colombia
**Jorge Luis Orozco**	Colombia	Valle del Lili Foundation
**Rio**	Colombia	University of Antioquia
**Alvaro Hernandez**	Costa Rica	University of Costa Rica
**Mohamed Salama**	Egypt	American University in Cairo
**Biniyam Ayele**	Ethiopia	University of Addis Ababa
**Yared Zenebe**	Ethiopia	University of Addis Ababa
**Alexis Brice**	France	ICM Institute
**Jean-Christophe Corvol**	France	ICM Institute
**Anastasia Illarionova**	Germany	Diseases
**Brit Mollenhauer**	Germany	University of Gottingen
**Christine Klein**	Germany	University of Luebeck
**Eva-Juliane Vollstedt**	Germany	University of Luebeck
**Katja Lohmann**	Germany	University of Luebeck
**Lara Mariah Lange**	Germany	University of Luebeck
**Manu Sharma**	Germany	University of Tuebingen
**Peter Heutink**	Germany	Diseases
**Thomas Gasser**	Germany	Hertie-Institute for Clinical Brain Research
**Zih-Hua Fang**	Germany	Diseases
**Albert Akpalu**	Ghana	University of Ghana
**Georgia Xiromerisiou**	Greece	University of Thessaly
**Leonidas Stefanis**	Greece	Academy of Athens
**Andrew Sobering**	Grenada	St. George's University
**Alex Medina**	Honduras	Hospital San Felipe
**Germaine Chan**	Hong Kong	Queen Elizabeth Hospital
**Nancy Ip**	Hong Kong	Technology
**Nelson Yuk-Fai Cheung**	Hong Kong	Queen Elizabeth Hospital
**Phillip Chan**	Hong Kong	Queen Elizabeth Hospital

**XiaoPu Zhou**	Hong Kong	Technology
**Asha Kishore**	India	Sciences & Technology
**Pramod Pal**	India	Neurosciences
**Roopa Rajan**	India	All India Institutes of Medical Sciences
**Rupam Borgohain**	India	Nizam's Institute Of Medical Sciences
**Enza Maria Valente**	Italy	University of Pavia, Italy
**Micol Avenali**	Italy	University of Pavia
**Tommaso Schirinzi**	Italy	University of Rome Tor Vergata
**Manabu Funayama**	Japan	Juntendo University
**Nobu Hattori**	Japan	Juntendo University
**Tomotaka Shiraishi**	Japan	Jikei University
**Tomotaka Shiraishi**	Japan	Jikei University
**Rejko Kruger**	Luxembourg	University of Luxembourg
**Ai Huey Tan**	Malaysia	University of Malaya
**Azlina Ahmad-Annuar**	Malaysia	University of Malaya
**Nor Azian Abdul Murad**	Malaysia	Centre
**Ibrahim**	Malaysia	Centre
**Shahrul Azmin**	Malaysia	Centre
**Shen-Yang Lim**	Malaysia	University of Malaya
**Wael Mohamed**	Malaysia	Centre
**Daniel Martinez**	Mexico	Monterrey Institute of Technology
**Violante**	Mexico	Neurosurgery
**Tim Anderson**	New Zealand	New Zealand Brain Research Institute
**Toni Pitcher**	New Zealand	
**Njideka Okubadejo**	Nigeria	University of Lagos
**Oluwadamilola Ojo**	Nigeria	University of Lagos
**Jan Aasly**	Norway	University of Trondheim
**Lasse Pihlstrøm**	Norway	Oslo University Hospital
**Shoaib Urrehman**	Pakistan	University of Science & Technology Bannu
**Mario Cornejo-Olivas**	Peru	National Institute of Neurological Sciences
**Angel Vinuela**	Puerto Rico	University of Puerto Rico
**Elena Iakovenko**	Russia	Research Center of Neurology
**Jia Nee Foo**	Singapore	Nanyang Technological University
**Soraya Bardien**	South Africa	Stellenbosch University
**Yun Joong Kim**	South Korea	Yonsei University
**Carrasco**	Spain	University College London
**Janet Hoenicka**	Spain	Sant Joan de Deu
**Sarah Elsadig**	Sudan	University of Khartoum
**Chin-Shien Lin**	Taiwan	National Taiwan University Hospital
**Ruey-Meei Robin Wu**	Taiwan	National Taiwan University Hospital
**Serena Wu**	Taiwan	Chang Gung University
**Yih-Ru Wu**	Taiwan	Chang Gung University
**Samia Ben Sassi**	Tunisia	Neurology
**Gencer Genc**	Turkey	Koc University
**Nazli Basak**	Turkey	Koc University
**Özgür Öztop Çakmak**	Turkey	Koc University
**Sibel Ertan**	Turkey	Koc University
**Alastair Noyce**	UK	Queen Mary University of London
**Camille Carroll**	UK	University Hospitals Plymouth
**Claire Bale**	UK	Parkinson's UK
**Clodagh Towns**	UK	University College London
**Henry Houlden**	UK	University College London

**Huw Morris**	UK	University College London
**John Hardy**	UK	University College London
**Joseph Callanan**	UK	Queen Mary University of London
**Kin Mok**	UK	University College London
**Manuela Tan**	UK	University College London
**Mie Rizig**	UK	University College London
**Nick Wood**	UK	University College London
**Nigel Williams**	UK	Cardiff University
**Olaitan Okunoye**	UK	University College London
**Patrick Lewis**	UK	Royal Veterinary College
**Rauan Kaiyrzanov**	UK	University College London
**Rimona Weil**	UK	University College London
**Simon Stott**	UK	Cure Parkinson's Trust
**Sumit Dey**	UK	Queen Mary University of London
**Alyssa O'Grady**	USA	The Michael J. Fox Foundation
**Bradford Casey**	USA	Michael J. Fox Foundation
**Caroline Pantazis**	USA	National Institutes of Health
**Claire Wegel**	USA	Indiana University
**Dan Vitale**	USA	Data Tecnica International
**Deborah Hall**	USA	Rush University
**Ejaz Shamim**	USA	Kaiser Permanente
**Faraz Faghri**	USA	Data Tecnica International / NIH
**Hampton Leonard**	USA	Data Tecnica International / NIH
**Hirotaka Iwaki**	USA	Data Tecnica International / NIH
**Ignacio Fernandez Mata**	USA	Cleveland Clinic
**Ignacio Juan Keller Sarmiento**	USA	Northwestern University
**Jeff Kim**	USA	National Institutes of Health
**Joshua Shulman**	USA	Baylor College of Medicine
**Justin C. Solle**	USA	The Michael J. Fox Foundation
**Karl Kieburtz**	USA	Beth Israel Deaconess Medical Center
**Ken Marek**	USA	The Michael J. Fox Foundation
**Lana Chahine**	USA	Michael J. Fox Foundation
**Laurel Screven**	USA	National Institutes of Health
**Lisa Shulman**	USA	University of Maryland
**Maggie Kuhl**	USA	The Michael J. Fox Foundation
**Marissa Dean**	USA	University of Alabama at Birmingham
**Mary Makarious**	USA	National Institutes of Health
**Miguel Inca**	USA	Cleveland Clinic
**Mike Nalls**	USA	Data Tecnica International
**Niccolo Mencacci**	USA	Northwestern University
**Roy Alcalay**	USA	Parkinson's Foundation
**Ruqaya Murtadha**	USA	National Institutes of Health
**Sara Bandres-Ciga**	USA	National Institutes of Health
**Schuyler Fox**	USA	Michael J. Fox Foundation
**Sohini Chowdhury**	USA	Michael J. Fox Foundation
**Steven Lubbe**	USA	Northwestern University
**Tao Xie**	USA	University of Chicago
**Tatiana Foroud**	USA	Indiana University School of Medicine
**Todd Sherer**	USA	Michael J. Fox Foundation
**Yeajin Song**	USA	Data Tecnica International
**Andrew Singleton**	USA	National Institutes of Health

**Bernadette Siddiqi**	USA	The Michael J. Fox Foundation
**Brian Fiske**	USA	The Michael J. Fox Foundation
**Cornelis Blauwendraat**	USA	National Institutes of Health
**Ekemini Riley**	USA	ASAP
**Duan Nguyen**	Vietnam	Hue University
**Toan Nguyen**	Vietnam	Hue University
**Masharip Atadzhanov**	Zambia	University of Zambia

Last update was 24, March 2020
IPDGC consortium members and affiliations:

United Kingdom: Alastair J Noyce (Preventive Neurology Unit, Wolfson Institute of Preventive Medicine, QMUL, London, UK and Department of Molecular Neuroscience, UCL, London, UK), Rauan Kaiyrzhanov (Department of Molecular Neuroscience, UCL Institute of Neurology, London, UK), Ben Middlehurst (Institute of Translational Medicine, University of Liverpool, Liverpool, UK), Demis A Kia (UCL Genetics Institute; and Department of Molecular Neuroscience, UCL Institute of Neurology, London, UK), Manuela Tan (Department of Clinical Neuroscience, University College London, London, UK), Henry Houlden (Department of Molecular Neuroscience, UCL Institute of Neurology, London, UK), Catherine S Storm (Department of Clinical and Movement Neurosciences, UCL Queen Square Institute of Neurology, London, UK), Huw R Morris (Department of Clinical Neuroscience, University College London, London, UK), Helene Plun-Favreau (Department of Molecular Neuroscience, UCL Institute of Neurology, London, UK), Peter Holmans (Biostatistics & Bioinformatics Unit, Institute of Psychological Medicine and Clinical Neuroscience, MRC Centre for Neuropsychiatric Genetics & Genomics, Cardiff, UK), John Hardy (Department of Molecular Neuroscience, UCL Institute of Neurology, London, UK), Daniah Trabzuni (Department of Molecular Neuroscience, UCL Institute of Neurology, London, UK; Department of Genetics, King Faisal Specialist Hospital and Research Centre, Riyadh, 11211 Saudi Arabia), John Quinn (Institute of Translational Medicine, University of Liverpool, Liverpool, UK), Vivien Bubb (Institute of Translational Medicine, University of Liverpool, Liverpool, UK), Kin Y Mok (Department of Molecular Neuroscience, UCL Institute of Neurology, London, UK), Kerri J. Kinghorn (Institute of Healthy Ageing, Research Department of Genetics, Evolution and Environment, University College London, London, UK), Nicholas W Wood (UCL Genetics Institute; and Department of Molecular Neuroscience, UCL Institute of Neurology, London, UK), Patrick Lewis (University of Reading, Reading, UK), Sebastian R Schreglmann (Department of Molecular Neuroscience, UCL Institute of Neurology, London, UK), Ruth Lovering (University College London, London, UK), Lea R'Bibo (Department of Molecular Neuroscience, UCL Institute of Neurology, London, UK), Claudia Manzoni (University of Reading, Reading, UK), Mie Rizig (Department of Molecular Neuroscience, UCL Institute of Neurology, London, UK), Mina Ryten (Department of Molecular Neuroscience, UCL Institute of Neurology, London, UK), Sebastian Guelfi (Department of Molecular Neuroscience, UCL Institute of Neurology, London, UK), Valentina Escott-Price (MRC Centre for Neuropsychiatric Genetics and Genomics, Cardiff University School of Medicine, Cardiff, UK), Viorica Chelban (Department of Molecular Neuroscience, UCL Institute of Neurology, London, UK), Thomas Foltynie (UCL Institute of Neurology, London, UK), Nigel Williams (MRC Centre for Neuropsychiatric Genetics and Genomics, Cardiff, UK), Karen E. Morrison (Faculty of Medicine, University of Southampton, UK), Carl Clarke (University of Birmingham, Birmingham, UK and Sandwell and West Birmingham Hospitals NHS Trust, Birmingham, UK), Kirsten Harvey (UCL School of Pharmacy, UK), Benjamin M Jacobs (Preventive Neurology Unit, Wolfson Institute of Preventive Medicine, QMUL, London, UK).

France: Alexis Brice (Institut du Cerveau et de la Moelle épinière, ICM, Inserm U 1127, CNRS, UMR 7225, Sorbonne Universités, UPMC University Paris 06, UMR S 1127, AP-HP, Pitié-Salpêtrière Hospital, Paris, France), Fabrice Danjou (Institut du Cerveau et de la Moelle épinière, ICM, Inserm U 1127, CNRS, UMR 7225, Sorbonne Universités, UPMC University Paris 06, UMR S 1127, AP-HP, Pitié-Salpêtrière Hospital, Paris, France), Suzanne Lesage (Institut du Cerveau et de la Moelle épinière, ICM, Inserm U 1127, CNRS, UMR 7225, Sorbonne Universités, UPMC University Paris 06, UMR S 1127, AP-HP, Pitié-Salpêtrière Hospital, Paris, France), Jean-Christophe Corvol (Institut du Cerveau et de la Moelle épinière, ICM, Inserm U 1127, CNRS, UMR 7225, Sorbonne Universités, UPMC University Paris 06, UMR

S 1127, Centre d'Investigation Clinique Pitié Neurosciences CIC-1422, AP-HP, Pitié-Salpêtrière Hospital, Paris, France), Maria Martinez (INSERM UMR 1220; and Paul Sabatier University, Toulouse, France),

Germany: Claudia Schulte (Department for Neurodegenerative Diseases, Hertie Institute for Clinical Brain Research, University of Tübingen, and DZNE, German Center for Neurodegenerative Diseases, Tübingen, Germany), Kathrin Brockmann (Department for Neurodegenerative Diseases, Hertie Institute for Clinical Brain Research, University of Tübingen, and DZNE, German Center for Neurodegenerative Diseases, Tübingen, Germany), Javier Simón-Sánchez (Department for Neurodegenerative Diseases, Hertie Institute for Clinical Brain Research, University of Tübingen, and DZNE, German Center for Neurodegenerative Diseases, Tübingen, Germany), Peter Heutink (DZNE, German Center for Neurodegenerative Diseases and Department for Neurodegenerative Diseases, Hertie Institute for Clinical Brain Research, University of Tübingen, Tübingen, Germany), Patrizia Rizzu (DZNE, German Center for Neurodegenerative Diseases), Manu Sharma (Centre for Genetic Epidemiology, Institute for Clinical Epidemiology and Applied Biometry, University of Tubingen, Germany), Thomas Gasser (Department for Neurodegenerative Diseases, Hertie Institute for Clinical Brain Research, and DZNE, German Center for Neurodegenerative Diseases, Tübingen, Germany), Susanne A. Schneider (Department of Neurology, Ludwig-Maximilians-University Munich, München, Germany)

United States of America: Mark R Cookson (Laboratory of Neurogenetics, National Institute on Aging, Bethesda, USA), Sara Bandres-Ciga (Laboratory of Neurogenetics, National Institute on Aging, Bethesda, MD, USA), Cornelis Blauwendraat (Laboratory of Neurogenetics, National Institute on Aging, Bethesda, MD, USA), David W. Craig (Department of Translational Genomics, Keck School of Medicine, University of Southern California, Los Angeles, USA), Kimberley Billingsley (Laboratory of Neurogenetics, National Institute on Aging, Bethesda, MD, USA), Mary B. Makarious (Laboratory of Neurogenetics, National Institute on Aging, Bethesda, MD, USA), Derek P. Narendra (Inherited Movement Disorders Unit, National pInstitute of Neurological Disorders and Stroke, Bethesda, MD, USA), Faraz Faghri (Laboratory of Neurogenetics, National Institute on Aging, Bethesda, USA; Department of Computer Science, University of Illinois at Urbana-Champaign, Urbana, IL, USA), J Raphael Gibbs (Laboratory of Neurogenetics, National Institute on Aging, National Institutes of Health, Bethesda, MD, USA), Dena G. Hernandez (Laboratory of Neurogenetics, National Institute on Aging, Bethesda, MD, USA), Kendall Van Keuren-Jensen (Neurogenomics Division, TGen, Phoenix, AZ USA), Joshua M. Shulman (Departments of Neurology, Neuroscience, and Molecular & Human Genetics, Baylor College of Medicine, Houston, Texas, USA; Jan and Dan Duncan Neurological Research Institute, Texas Children's Hospital, Houston, Texas, USA), Hirotaka Iwaki (Laboratory of Neurogenetics, National Institute on Aging, Bethesda, MD, USA), Hampton L. Leonard (Laboratory of Neurogenetics, National Institute on Aging, Bethesda, MD, USA), Mike A. Nalls (Laboratory of Neurogenetics, National Institute on Aging, Bethesda, USA; CEO/Consultant Data Tecnica International, Glen Echo, MD, USA), Laurie Robak (Baylor College of Medicine, Houston, Texas, USA), Jose Bras (Center for Neurodegenerative Science, Van Andel Research Institute, Grand Rapids, Michigan, USA), Rita Guerreiro (Center for Neurodegenerative Science, Van Andel Research Institute, Grand Rapids, Michigan, USA), Steven Lubbe (Ken and Ruth Davee Department of Neurology and Simpson Querrey Center for Neurogenetics, Northwestern University Feinberg School of Medicine, Chicago, IL, USA), Bernabe I. Bustos (Ken and Ruth Davee Department of Neurology and Simpson Querrey Center for Neurogenetics, Northwestern University Feinberg School of Medicine, Chicago, IL, USA), Timothy Troycoco (National Institutes of Health, USA), Steven Finkbeiner (Departments of Neurology and Physiology, University of California, San Francisco; Gladstone Institute of Neurological Disease; Taube/Koret Center for Neurodegenerative Disease Research, San Francisco, CA, USA), Niccolo E. Mencacci (Northwestern University Feinberg School of Medicine, Chicago, IL, USA), Codrin Lungu (National Institutes of Health Division of Clinical Research, NINDS, National Institutes of

Health, Bethesda, MD, USA), Andrew B Singleton (Laboratory of Neurogenetics, National Institute on Aging, Bethesda, MD, USA), Sonja W. Scholz (Neurodegenerative Diseases Research Unit, National Institute of Neurological Disorders and Stroke, Bethesda, MD, USA), Xylena Reed (Laboratory of Neurogenetics, National Institute on Aging, Bethesda, MD, USA). Roy N. Alcalay (Department of Neurology, College of Physicians and Surgeons, Columbia University Medical Center, New York, NY, USA, Taub Institute for Research on Alzheimer's Disease and the Aging Brain, College of Physicians and Surgeons, Columbia University Medical Center, New York, NY, USA), Zbigniew K. Wszolek (Department of Neurology, Mayo Clinic Jacksonville, FL, USA), Ryan J. Uitti (Department of Neurology, Mayo Clinic Jacksonville, FL, USA), Owen A. Ross (Departments of Neuroscience & Clinical Genomics, Mayo Clinic Jacksonville, FL, USA), Francis P. Grenn (Laboratory of Neurogenetics, National Institute on Aging, Bethesda, MD, USA), Anni Moore (Laboratory of Neurogenetics, National Institute on Aging, Bethesda, MD, USA), Vanessa Pitz (Laboratory of Neurogenetics, National Institute on Aging, Bethesda, MD, USA).

Canada: Ziv Gan-Or (Montreal Neurological Institute and Hospital, Department of Neurology & Neurosurgery, Department of Human Genetics, McGill University, Montréal, QC, H3A 0G4, Canada), Guy A. Rouleau (Montreal Neurological Institute and Hospital, Department of Neurology & Neurosurgery, Department of Human Genetics, McGill University, Montréal, QC, H3A 0G4, Canada), Lynne Krohn (Montreal Neurological Institute and Hospital, Department of Neurology & Neurosurgery, Department of Human Genetics, McGill University, Montréal, QC, H3A 0G4, Canada), Kheireddin Mufti (Montreal Neurological Institute and Hospital, Department of Neurology & Neurosurgery, Department of Human Genetics, McGill University, Montréal, QC, H3A 0G4, Canada),

The Netherlands: Jacobus J van Hilten (Department of Neurology, Leiden University Medical Center, Leiden, Netherlands), Johan Marinus (Department of Neurology, Leiden University Medical Center, Leiden, Netherlands)

Spain: Astrid D. Adarmes-Gómez (Instituto de Biomedicina de Sevilla (IBiS), Hospital Universitario Virgen del Rocío/CSIC/Universidad de Sevilla, Seville), Miquel Aguilar (Fundació Docència i Recerca Mútua de Terrassa and Movement Disorders Unit, Department of Neurology, University Hospital Mutua de Terrassa, Terrassa, Barcelona.), Ignacio Alvarez (Fundació Docència i Recerca Mútua de Terrassa and Movement Disorders Unit, Department of Neurology, University Hospital Mutua de Terrassa, Terrassa, Barcelona), Victoria Alvarez (Hospital Universitario Central de Asturias, Oviedo), Francisco Javier Barrero (Hospital Universitario San Cecilio de Granada, Universidad de Granada), Jesús Alberto Bergareche Yarza (Instituto de Investigación Sanitaria Biodonostia, San Sebastián), Inmaculada Bernal-Bernal (Instituto de Biomedicina de Sevilla (IBiS), Hospital Universitario Virgen del Rocío/CSIC/Universidad de Sevilla, Seville), Marta Blazquez (Hospital Universitario Central de Asturias, Oviedo), Marta Bonilla-Toribio (Instituto de Biomedicina de Sevilla (IBiS), Hospital Universitario Virgen del Rocío/CSIC/Universidad de Sevilla, Seville), Juan A. Botía (Universidad de Murcia, Murcia), María Teresa Boungiorno (Fundació Docència i Recerca Mútua de Terrassa and Movement Disorders Unit, Department of Neurology, University Hospital Mutua de Terrassa, Terrassa, Barcelona), Dolores Buiza-Rueda (Instituto de Biomedicina de Sevilla (IBiS), Hospital Universitario Virgen del Rocío/CSIC/Universidad de Sevilla, Seville), Ana Cámara (Hospital Clinic de Barcelona), Fátima Carrillo (Instituto de Biomedicina de Sevilla (IBiS), Hospital Universitario Virgen del Rocío/CSIC/Universidad de Sevilla, Seville), Mario Carrión-Claro (Instituto de Biomedicina de Sevilla (IBiS), Hospital Universitario Virgen del Rocío/CSIC/Universidad de Sevilla, Seville), Debora Cerdan (Hospital General de Segovia, Segovia), Jordi Clarimón (Memory Unit, Department of Neurology, IIB Sant Pau, Hospital de la Santa Creu i Sant Pau, Universitat Autònoma de Barcelona and Centro de Investigación Biomédica en Red en Enfermedades Neurodegenerativas (CIBERNED), Madrid), Yaroslau Compta (Hospital Clinic de Barcelona), Monica Diez-Fairen (Fundació

Docència i Recerca Mútua de Terrassa and Movement Disorders Unit, Department of Neurology, University Hospital Mutua de Terrassa, Terrassa, Barcelona.), Oriol Dols-Icardo (Memory Unit, Department of Neurology, IIB Sant Pau, Hospital de la Santa Creu i Sant Pau, Universitat Autònoma de Barcelona, Barcelona, and Centro de Investigación Biomédica en Red en Enfermedades Neurodegenerativas (CIBERNED) Madrid), Oriol de Fabregues (Movement Disorders Unit, Neurology Department, University Hospital Vall d'Hebron Barcelona), Pilar Sanz Cartagena (Hospital de Mataro Consorci Sanitari del Maresme Mataro), Jacinto Duarte (Hospital General de Segovia, Segovia), Raquel Duran (Centro de Investigacion Biomedica, Universidad de Granada, Granada), Francisco Escamilla-Sevilla (Hospital Universitario Virgen de las Nieves, Instituto de Investigación Biosanitaria de Granada, Granada), Mario Ezquerra (Hospital Clinic de Barcelona), Cici Feliz (Departmento de Neurologia, Instituto de Investigación Sanitaria Fundación Jiménez Díaz, Madrid, Spain), Manel Fernández (Hospital Clinic de Barcelona), Rubén Fernández-Santiago (Hospital Clinic de Barcelona), Ciara Garcia (Hospital Universitario Central de Asturias, Oviedo), Pedro García-Ruiz (Instituto de Investigación Sanitaria Fundación Jiménez Díaz, Madrid), Pilar Gómez-Garre (Instituto de Biomedicina de Sevilla (IBiS), Hospital Universitario Virgen del Rocío/CSIC/Universidad de Sevilla, Seville), Maria Jose Gomez Heredia (Hospital Universitario Virgen de la Victoria, Malaga), Isabel Gonzalez-Aramburu (Hospital Universitario Marqués de Valdecilla-IDIVAL, Santander), Ana Gorostidi Pagola (Instituto de Investigación Sanitaria Biodonostia, San Sebastián), Janet Hoenicka (Institut de Recerca Sant Joan de Déu, Barcelona), Jon Infante (Hospital Universitario Marqués de Valdecilla-IDIVAL and University of Cantabria, Santander, and Centro de Investigación Biomédica en Red en Enfermedades Neurodegenerativas (CIBERNED), Silvia Jesús (Instituto de Biomedicina de Sevilla (IBiS), Hospital Universitario Virgen del Rocío/CSIC/Universidad de Sevilla, Seville), Adriano Jimenez-Escrig (Hospital Universitario Ramón y Cajal, Madrid), Jaime Kulisevsky (Movement Disorders Unit, Department of Neurology, IIB Sant Pau, Hospital de la Santa Creu i Sant Pau, Universitat Autònoma de Barcelona, Barcelona, and Centro de Investigación Biomédica en Red en Enfermedades Neurodegenerativas (CIBERNED)), Miguel A. Labrador-Espinosa (Instituto de Biomedicina de Sevilla (IBiS), Hospital Universitario Virgen del Rocío/CSIC/Universidad de Sevilla, Seville), Jose Luis Lopez-Sendon (Hospital Universitario Ramón y Cajal, Madrid), Adolfo López de Munain Arregui (Instituto de Investigación Sanitaria Biodonostia, San Sebastián), Daniel Macias (Instituto de Biomedicina de Sevilla (IBiS), Hospital Universitario Virgen del Rocío/CSIC/Universidad de Sevilla, Seville), Irene Martínez Torres (Department of Neurology, Instituto de Investigación Sanitaria La Fe, Hospital Universitario y Politécnico La Fe, Valencia), Juan Marín (Movement Disorders Unit, Department of Neurology, IIB Sant Pau, Hospital de la Santa Creu i Sant Pau, Universitat Autònoma de Barcelona, Barcelona, and Centro de Investigación Biomédica en Red en Enfermedades Neurodegenerativas (CIBERNED)), Maria Jose Marti (Hospital Clinic Barcelona), Juan Carlos Martínez-Castrillo (Instituto Ramón y Cajal de Investigación Sanitaria, Hospital Universitario Ramón y Cajal, Madrid), Carlota Méndez-del-Barrio (Instituto de Biomedicina de Sevilla (IBiS), Hospital Universitario Virgen del Rocío/CSIC/Universidad de Sevilla, Seville), Manuel Menéndez González (Hospital Universitario Central de Asturias, Oviedo), Marina Mata (Department of Neurology, Hospital Universitario Infanta Sofía, Madrid, Spain), Adolfo Mínguez (Hospital Universitario Virgen de las Nieves, Granada, Instituto de Investigación Biosanitaria de Granada), Pablo Mir (Instituto de Biomedicina de Sevilla (IBiS), Hospital Universitario Virgen del Rocío/CSIC/Universidad de Sevilla, Seville), Elisabet Mondragon Rezola (Instituto de Investigación Sanitaria Biodonostia, San Sebastián), Esteban Muñoz (Hospital Clinic Barcelona), Javier Pagonabarraga (Movement Disorders Unit, Department of Neurology, IIB Sant Pau, Hospital de la Santa Creu i Sant Pau, Universitat Autònoma de Barcelona, Barcelona, and Centro de Investigación Biomédica en Red en Enfermedades Neurodegenerativas (CIBERNED)), Pau Pastor (Fundació Docència i Recerca Mútua de Terrassa and Movement Disorders Unit, Department of Neurology, University Hospital Mutua de Terrassa, Terrassa, Barcelona.), Francisco Perez Errazquin (Hospital Universitario Virgen de la Victoria, Malaga), Teresa

Periñán-Tocino (Instituto de Biomedicina de Sevilla (IBiS), Hospital Universitario Virgen del Rocío/CSIC/Universidad de Sevilla, Seville), Javier Ruiz-Martínez (Hospital Universitario Donostia, Instituto de Investigación Sanitaria Biodonostia, San Sebastián), Clara Ruz (Centro de Investigacion Biomedica, Universidad de Granada, Granada), Antonio Sanchez Rodriguez (Hospital Universitario Marqués de Valdecilla-IDIVAL, Santander), María Sierra (Hospital Universitario Marqués de Valdecilla-IDIVAL, Santander), Esther Suarez-Sanmartin (Hospital Universitario Central de Asturias, Oviedo), Cesar Tabernero (Hospital General de Segovia, Segovia), Juan Pablo Tartari (Fundació Docència i Recerca Mútua de Terrassa and Movement Disorders Unit, Department of Neurology, University Hospital Mutua de Terrassa, Terrassa, Barcelona), Cristina Tejera-Parrado (Instituto de Biomedicina de Sevilla (IBiS), Hospital Universitario Virgen del Rocío/CSIC/Universidad de Sevilla, Seville), Eduard Tolosa (Hospital Clinic Barcelona), Francesc Valldeoriola (Hospital Clinic Barcelona), Laura Vargas-González (Instituto de Biomedicina de Sevilla (IBiS), Hospital Universitario Virgen del Rocío/CSIC/Universidad de Sevilla, Seville), Lydia Vela (Department of Neurology, Hospital Universitario Fundación Alcorcón, Madrid), Francisco Vives (Centro de Investigacion Biomedica, Universidad de Granada, Granada), Pilar Sanz Cartagena (Hospital de Mataro Consorci Sanitari del Maresme, Mataro), Oriol de Fabregues (Movement Disorders Unit, Neurology Department, University Hospital Vall d'Hebron, Barcelona).

Austria: Alexander Zimprich (Department of Neurology, Medical University of Vienna, Austria)

Norway: Lasse Pihlstrom (Department of Neurology, Oslo University Hospital, Oslo, Norway), Mathias Toft (Department of Neurology and Institute of Clinical Medicine, Oslo University Hospital, Oslo, Norway)

Estonia: Pille Taba (Department of Neurology and Neurosurgery, University of Tartu, Tartu, Estonia)

Australia: Sulev Koks (Centre for Molecular Medicine and Innovative Therapeutics, Murdoch University, Murdoch, 6150, Perth, Western Australia; The Perron Institute for Neurological and Translational Science, Nedlands, 6009, Perth, Western Australia)

Israel: Sharon Hassin-Baer (The Movement Disorders Institute, Department of Neurology and Sagol Neuroscience Center, Chaim Sheba Medical Center, Tel-Hashomer, 5262101, Ramat Gan, Israel, Sackler Faculty of Medicine, Tel Aviv University, Tel Aviv, Israel)

Finland: Kari Majamaa (Institute of Clinical Medicine, Department of Neurology, University of Oulu, Oulu, Finland; Department of Neurology and Medical Research Center, Oulu University Hospital, Oulu, Finland), Ari Siitonen (Institute of Clinical Medicine, Department of Neurology, University of Oulu, Oulu, Finland; Department of Neurology and Medical Research Center, Oulu University Hospital, Oulu, Finland), Pentti Tienari (Clinical Neurosciences, Neurology, University of Helsinki, Helsinki, Finland, Helsinki University Hospital, Helsinki, Finland)

Nigeria: Njideka U. Okubadejo (University of Lagos, Lagos State, Nigeria), Oluwadamilola O. Ojo (University of Lagos, Lagos State, Nigeria),

Kazakhstan: Coordinator - Rauan Kaiyrzhanov (Department of Molecular Neuroscience, UCL Institute of Neurology, London, UK), Nazira Zharkinbekova (South Kazakhstan Medical Academy, Shymkent, Kazakhstan), Vadim Akhmetzhanov (Astana Medical University, Astana Kazakhstan), Gulnaz Kaishybayeva (Scientific and practical center “Institute of neurology named after Smagul Kaishibayev”,

Almaty, Kazakhstan), Altynay Karimova (Scientific and practical center “Institute of neurology named after Smagul Kaishibayev”, Almaty, Kazakhstan).
Ireland: Timothy L. Lynch (The Dublin Neurological Institute at the Mater Misericordiae University Hospital, Dublin, Ireland & School of Medicine and Medical Science, University College Dublin, Dublin, Ireland).
